# The *α*_2_*β*_1_ integrin mediates the malignant phenotype on type I collagen in pancreatic cancer cell lines

**DOI:** 10.1038/sj.bjc.6603088

**Published:** 2006-04-04

**Authors:** J J Grzesiak, M Bouvet

**Affiliations:** 1 Department of Surgery (112-E), University of California, Veterans Affairs San Diego Healthcare System, 3350 La Jolla Village Drive, San Diego, CA 92161, USA

**Keywords:** laminin, fibronectin, extracellular matrix, migration, adhesion

## Abstract

Pancreatic cancer is characterised by a hallmark desmoplastic response that includes upregulated expression of the extracellular matrix, and type I collagen in particular. Recent studies indicate that pancreatic cancer cells stimulate type I collagen synthesis in adjacent stellate cells, and that this upregulated type I collagen expression promotes the malignant phenotype in tumour cells as defined by increased proliferation, resistance to chemically induced apoptosis, and increased tumorigenesis. The integrin specificity of this interaction between type I collagen and tumour cells was not identified, however. In the present study, we examined eight pancreatic cancer cell lines for adhesion, proliferation, and migration, on types I and IV collagen, fibronectin, laminin, and vitronectin, as well as integrin expression. Our results indicate, for the overwhelming majority of cell lines, that type I collagen promotes the strongest adhesion, proliferation, and migration relative to the other substrates tested. Utilising function-blocking monoclonal antibodies directed against particular integrin subunits in cell adhesion and migration inhibition assays, we demonstrate further that the malignant phenotype on type I collagen is mediated specifically by the *α*_2_*β*_1_ integrin. These results identify *α*_2_*β*_1_ integrin-mediated adhesion to type I collagen as a potential therapeutic target in the treatment of pancreatic cancer.

Pancreatic ductal adenocarcinoma is thought to result from a progressive accumulation of mutations in such genes as K-ras, CDKN2A, p53, BRCA2, p16^4ink^, and SMAD4 (Bardeesy and DePinho, 2002). The SMAD4 mutations, in particular, which result in the constitutive activation of transforming growth factor *β*_1_ (TGF*β*_1_) signalling, are generally considered to be responsible for the hallmark desmoplastic response, which includes upregulated expression of the extracellular matrix (ECM), and type I collagen in particular (Mollenhauer *et al*, 1987; Lohr *et al*, 1994; Shimoyama *et al*, 1995; Linder *et al*, 2001; Bardeesy and DePinho, 2002; Menke and Adler, 2002; Tempia-Caliera *et al*, 2002; Iacobuzio-Donahue *et al*, 2003a, 2003b).

We have previously shown that *α*_2_*β*_1_ integrin-mediated adhesion on type I collagen promotes the malignant phenotype in FG pancreatic cells, as defined by increased proliferation and haptokinetic cell migration, downregulated expression and localisation of E-cadherin and *β*-catenin in cell–cell contacts, increased phosphorylation of GSK3*β* and PKB/Akt, and downregulated expression of PTHrP, IL-6, and IL-8 compared to fibronectin, type IV collagen, laminin, or vitronectin (Grzesiak *et al*, 2004, 2005a). These results are in agreement with previous studies demonstrating in Panc-1, BxPC-3, and PaTu8988s pancreatic cancer cells that type I collagen downregulates E-cadherin expression, resulting in increased proliferation and migration compared to fibronectin (Menke *et al*, 2001). Recently, we extended our initial observations in FG cells by demonstrating similar phenotypic differences in BxPC-3, Colo-357, and CFPAC cells on type I collagen compared to fibronectin; that is, increased haptokinetic cell migration, downregulated expression and localisation of E-cadherin and *β*-catenin in disrupted cell–cell contacts, increased phosphorylation of GSK3 and PKB/Akt, and decreased expression of PTHrP, IL-6, and IL-8. Furthermore, functional studies with pharmacological inhibitors for GSK3 and PKB/Akt suggest that these signalling effectors are involved in the mechanism of *α*_2_*β*_1_ integrin-mediated regulation of the malignant phenotype in FG cells (Grzesiak *et al*, 2006).

Recent studies from several other laboratories have also converged on the hypothesis that type I collagen, in particular, plays an active role *in vitro* and *in vivo* in the pathophysiology of pancreatic cancer (Armstrong *et al*, 2004; Bachem *et al*, 2005). Specifically, these studies demonstrated *in vitro* that pancreatic cancer cell lines stimulated the production of type I collagen from adjacent stellate cells, which resulted in increased cancer cell proliferation and resistance to chemically induced apoptosis. *In vivo*, these studies also showed that type I collagen expression was closely associated with both the cancer and stellate cells, and that introduction of stellate cells along with the tumour cells increased tumorigenesis in nude mice. The integrin specificity of this interaction between tumour cells and type I collagen was not identified, however.

In the present study, we surveyed a broad range of pancreatic cancer cell lines from various tumour grades, origins, and genetic aberrations, for relative integrin expression, as well as adhesion, proliferation, and haptokinetic migration on types I and IV collagen, fibronectin, laminin, and vitronectin. Our results indicate strong *α*_2_*β*_1_ integrin-mediated type I collagen adhesion, proliferation, and migration for all cell lines, except MiaPaCa-2, which does not express the *α*_2_*β*_1_ collagen-binding integrin. These results collectively suggest an active and dynamic role for the ECM, and type I collagen in particular, in the growth, progression, and metastasis of pancreatic cancer, and identify the *α*_2_*β*_1_ integrin as a potential therapeutic target in the treatment of this devastating disease.

## MATERIALS AND METHODS

### Cells

Seven human pancreatic adenocarcinoma cell lines, Capan-1, CFPAC, Colo-357, AsPC-1, BxPC-3, MiaPaCa-2, and Panc-1 cells were obtained from the American Type Culture Collection (Rockville, MD, USA). FG cells are a fast-growing, metastatic variant of the Colo-357 cell line (Vezeridis *et al*, 1990), and were the generous gift from Dr S Silletti. The cell lines were cultured in Dulbecco's modified Eagle's medium (DMEM) supplemented with 10% foetal bovine serum (FBS) in a humidified atmosphere containing 5% CO_2_ at 37°C.

### Antibodies

The function-blocking monoclonal antibodies directed against particular integrin subunits and integrins used in these studies have been described (Cheresh and Spiro, 1987; Wayner and Carter, 1987; Sonnenberg *et al*, 1988; Wayner *et al*, 1988, 1993; Fabbri *et al*, 1996), and include FB12 (*α*_1_), P1E6 (*α*_2_), P1B5 (*α*_3_), P1D6 (*α*_5_), GoH3 (*α*_6_), P5D2 (*β*_1_), LM609 (*α*_v_*β*_3_), P1F6 (*α*_v_*β*_5_), and ASC-3 (*β*_4_) (Chemicon International, Temecula, CA, USA). For immunoprecipitation (IP) experiments, we used the monoclonal antibodies described above along with rabbit polyclonal antisera against the *α*_5_ integrin subunit, which has also been described previously (Ruoslahti and Pierschbacher, 1987).

### Cell adhesion assays

Cell adhesion assays were performed as described previously (Ruoslahti *et al*, 1982). Briefly, 5 × 10^4^ cells, serum-starved 24 h prior to assay, in 100 *μ*l of serum-free DMEM supplemented with 1 mg ml^−1^ bovine serum albumin (BSA) were added to each well of nontissue culture-treated 96-well microtitre plates (Becton Dickinson, Franklin Lakes, NJ, USA) that were previously coated overnight at room temperature with serial dilutions of purified ECM proteins, beginning with 100 *μ*g ml^−1^ through 0.79 *μ*g ml^−1^, and blocked with 1 mg ml^−1^ BSA in phosphate-buffered saline (PBS) for 1 h at 37°C. Extracellular matrix proteins included bovine types I and IV collagen, human fibronectin, mouse laminin, and human vitronectin (Chemicon International). Plates were then incubated for 1 h at 37°C, the unattached cells were removed, and the attached cells were fixed with 3% paraformaldehyde in PBS, stained with 0.5% toluidine blue in 3.7% formaldehyde, solubilised with 20% sodium dodecyl sulphate, and the absorbance at 595 nm was measured using a vertical pathway spectrophotometer.

### Inhibition of cell adhesion assays

Inhibition of cell adhesion assays were performed as described previously (Grzesiak *et al*, 1992, 2005a). Briefly, 96-well microtitre plates were previously coated with type I collagen (5 *μ*g ml^−1^) and unbound sites blocked with 1 mg ml^−1^ BSA as described above. Then, 5 × 10^4^ FG or CFPAC cells, or 2.5 × 10^4^ AsPC-1 or BxPC-3 cells were added to each well in serum-free DMEM supplemented with 1 mg ml^−1^ BSA. Purified monoclonal antibodies were added at a final concentration of 25 *μ*g ml^−1^ in the serum-free medium described above. After 45 min at 37°C, media were removed; attached cells were fixed, stained, destained, solubilised, and quantified as described above.

### Migration assays

Migration assays were conducted using the modified Boyden chamber as described previously (Grzesiak *et al*, 1992, 2005a). Briefly, the chamber consists of two compartments separated by a filter, and migration was measured by counting the number of cells crossing the membrane through pores of defined size. Lower chambers were filled with serum-free DMEM (Invitrogen, Carlsbad, CA, USA) supplemented with 1 mg ml^−1^ BSA. Pore polycarbonate membrane filters (8 *μ*M) (Neuro Probe Inc., Gaithersburg, MD, USA) that were coated with either type I collagen, type IV collagen, fibronectin, laminin, or vitronectin, each at 5 *μ*g ml^−1^, were then placed on top of the lower chambers, and the upper chambers were secured in place. Upper chambers were filled with 5 × 10^4^ FG, AsPC-1, MiaPaCa-2, BxPC-3, or CFPAC cells that were serum-starved 24 h prior to assay, in the same media described above. Lower chamber final volumes were 30 *μ*l and the upper chambers were 50 *μ*l. The entire apparatus was then incubated for 24 h at 37°C. After the incubation period, the filters were fixed in methanol and stained with 0.5% toluidine blue in 3.7% formaldehyde. Excess stain was washed away with water, the attached cells on the upper side of the filters were mechanically removed using wet, cotton-tipped applicators, and the migratory cells on the underside of the filters were quantitated by counting four high-powered fields (× 100 magnification) per well using an inverted light microscope (Olympus BH 2).

### Inhibition of cell migration assays

In some experiments, migration assays were conducted as described above, with the addition of function-blocking monoclonal antibodies directed against specific integrin subunits to the upper chamber at the time cells were added. Final antibody concentrations were 50 *μ*g ml^−1^ for FB12 (*α*_1_), P1E6 (*α*_2_), and P1B5 (*α*_3_), and 25 *μ*g ml^−1^ for P5D2 (*β*_1_). Type I collagen-coating concentrations were 5 *μ*g ml^−1^ for each cell line. Cells (5 × 10^4^) were added to each well, except for BxPC-3 cells, which were used at 2.5 × 10^4^ cells well^−1^.

### Immunoprecipitations

To perform IPs, antibodies (4 *μ*g) were adsorbed for 2 h at room temperature onto 25 *μ*l packed anti-mouse IgG–agarose, anti-rat IgG–agarose or protein A–sepharose (Sigma, St Louis, MO, USA) in IP wash buffer (50 mM Tris-Hcl, pH 7.5, 0.5 M NaCl, 1 mM MgCl_2_, 1 mM CaCl_2_, and 0.1% Tween-20). Antibody-coated beads were washed and 400 *μ*g protein from cell-surface-biotinylated cell lysates was added in IP wash buffer. Protein content was determined using the modified Bradford reagent according to the manufacturer's instructions (BioRad, Hercules, CA, USA). Pancreatic cancer cell lines were cell surface biotinylated using EZ-Link™ Sulfo-NHS-LC-Biotin (sulfosuccinimidyl-6-(biotinamido) hexanoate) according to the manufacturer's instructions (Pierce Biotechnology Inc., Rockford, IL, USA) as described previously (Grzesiak *et al*, 2005b). Cell lysates were prepared as previously described, except ethylenediaminetetraacetic acid was removed and replaced with 1 mM CaCl_2_ and 1 mM MgCl_2_ (Grzesiak *et al*, 2004, 2005a, 2005b). After incubation overnight at 4°C, the beads were washed six times with IP wash buffer, 50 *μ*l 2 × NU–PAGE sample buffer was added (Invitrogen, Carlsbad, CA, USA) samples were incubated 15 min at 70°C, and 25 *μ*l of sample was carefully loaded, separated on 12% NU–PAGE gels under nonreducing conditions, and transferred to nitrocellulose. After blocking with 3% BSA in PBS/0.1% Tween-20 overnight at 4°C, membranes were incubated with horseradish peroxidase-conjugated streptavidin (1 : 25 000 dilution; Pierce Biotechnology Inc.) for 30 min at room temperature, followed by washing, and detection of peroxidase activity using chemiluminescence according to the manufacturer's instructions (Amersham Biosciences, Little Chalfont, UK).

### Proliferation assays

**Figure 1:**
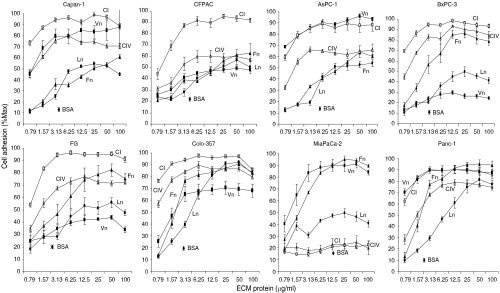
Type I collagen promotes maximal adhesion of pancreatic cancer cell lines compared to other ECM proteins. Cell adhesion assays were conducted as described in Materials and Methods with Capan-1, CFPAC, Colo-357, FG, AsPC-1, BxPC-3, MiaPaCa-2, and Panc-1 cells. CI, □ – type I collagen; CIV, Δ – type IV collagen; Fn, ▴ – fibronectin; Ln, • – laminin; and Vn, ▪ – vitronectin. Results are expressed as the mean±s.e.m. from two independent experiments conducted in duplicate. Nonspecific adhesion to BSA±s.e.m. is also noted for each cell line. 100%=maximal absorbance at 595 nm for each cell line and each set of ECM proteins.

The 96-well polystyrene culture dishes, not treated for tissue culture, were coated with type I collagen, type IV collagen, fibronectin, laminin, or vitronectin at the following concentrations: for AsPC-1 cells – types I and IV collagen at 5 *μ*g ml^−1^, fibronectin at 25 *μ*g ml^−1^, laminin at 15 *μ*g ml^−1^, and vitronectin at 5 *μ*g ml^−1^; for CFPAC cells – types I and IV collagen at 5 *μ*g ml^−1^, fibronectin and laminin at 25 *μ*g ml^−1^, and vitronectin at 10 *μ*g ml^−1^; for BxPC-3 cells – types I and IV collagen at 5 *μ*g ml^−1^, and fibronectin, laminin, and vitronectin each at 15 *μ*g ml^−1^; for MiaPaCa-2 cells – types I and IV collagen at 10 *μ*g ml^−1^, fibronectin at 25 *μ*g ml^−1^, laminin at 15 *μ*g ml^−1^, and vitronectin at 10 *μ*g ml^−1^; and for FG cells – types I and IV collagen at 5 *μ*g ml^−1^, laminin at 10 *μ*g ml^−1^, fibronectin at 25 *μ*g ml^−1^, and vitronectin at 10 *μ*g ml^−1^. These coating concentrations promote maximal adhesion for each cell line as shown in Figure 1. Twenty-four hour, serum-starved pancreatic cancer cells (5 × 10^3^ well^−1^) were cultured under serum-free conditions on these ECM substrata over a 4-day time course. At the indicated time points, triplicate proliferation determinations were quantified by measuring the absorbance at 450 nm and subtracting the value obtained for each cell line on each ECM substrate at initial seeding using CellTiter 96 Aqueous One Solution Cell Proliferation Assay™ reagent according to the manufacturer's instructions (Promega, Madison, WI, USA). This reagent is composed of a novel tetrazolium compound (methyl-*p*-tolyl sulfide), and an electron coupling reagent, phenazine ethosulphate.

### Statistics

Statistical significance (*P*<0.05) was determined using two-tailed Student's *t*-tests.

## RESULTS

### Type I collagen promotes maximal cell adhesion in pancreatic cancer cell lines

We first tested the panel of commonly used pancreatic cancer cell lines shown in Table 1, which covers all tumour grades and genetic mutations, and includes cell lines derived from primary tumours, ascites, and liver and lymph node metastases, in serum-free adhesion assays on the stromal ECM proteins, type I collagen, fibronectin, and vitronectin, and the basement membrane proteins, type IV collagen and laminin. Figure 1 demonstrates that in seven of eight cell lines tested, type I collagen promoted the strongest cell attachment relative to the other ECM proteins, and did so at much lower concentrations compared to the other ECM proteins tested. Only MiaPaCa-2 cells did not attach to type I collagen, nor did they attach to type IV collagen, even at the highest coating concentrations. Attachment to fibronectin was also quite strong in all the cell lines, except for the grade 1 tumour cell lines, Capan-1 and CFPAC, and the grade 2 cell line, AsPC-1, which showed only weak to moderate attachment. Laminin adhesion was moderate to weak in all cell lines tested. Adhesion of pancreatic cancer cell lines on vitronectin was the most variable. Relative to the other ECM proteins, FG, Colo-357, BxPC-3, and CFPAC cell adhesion to vitronectin was limited, while AsPC-1, Capan-1, Panc-1, and MiaPaCa-2 adhesion to vitronectin was very strong. A trend towards increased vitronectin adhesion with increasing tumour grade was apparent, with both grade 3-derived cell lines, MiaPaCa-2 and Panc-1, attaching strongly to vitronectin. Relative to the other cell lines, BxPC-3, CFPAC, and Panc-1 exhibited the strongest adhesion, regardless of substrate (unpublished observations).

### Type I collagen promotes maximal proliferation of pancreatic cancer cell lines

**Figure 2:**
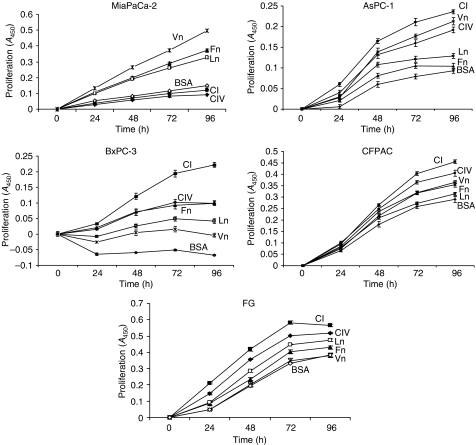
Type I collagen promotes maximal pancreatic cancer cell proliferation. The 96-well polystyrene culture dishes, not treated for tissue culture, were coated with type I collagen, type IV collagen, fibronectin, laminin, or vitronectin as described in Materials and Methods. Twenty-four hour, serum-starved MiaPaCa-2, AsPC-1, BxPC-3, CFPAC, and FG cells (5 × 10^3^ well^−1^) were cultured under serum-free conditions on the indicated ECM substrates over a 4-day time course. At the indicated time points, triplicate determinations were quantified for each cell line on each substrate by measuring the absorbance at 450 nm and subtracting the value obtained at initial seeding (*T*=0) using CellTiter 96 Aqueous One Solution Cell Proliferation Assay™ reagent according to the manufacturer's instructions (Promega, Madison, WI, USA). Data presented represent the mean±s.e.m. from two independent experiments.

We next examined the differential effect of the ECM on pancreatic cancer cell proliferation. Using ECM protein concentrations that promoted maximal cell adhesion for each of the substrates with five representative cell lines still representing all three tumour grades, we conducted 96 h proliferation assay time-course studies under serum-free conditions. All cell lines tested grew well throughout the 4-day time course, even in the absence of serum, as long as the cells were given an ECM substrate to which they could attach. Figure 2 shows a strong direct correlation between pancreatic cancer cell adhesion and proliferation, with AsPC-1, BxPC-3, CFPAC, and FG cells demonstrating maximal proliferation on type I collagen relative to the other ECM proteins examined. MiaPaCa-2 cells, by contrast, showed essentially no proliferation on either type I or type IV collagen. Visual inspection confirmed that MiaPaCa-2 cells were found as nonadherent floating aggregates when cultured on either of the collagens (unpublished observations).

### Type I collagen promotes maximal haptokinetic cell migration in pancreatic cancer cell lines

**Figure 3:**
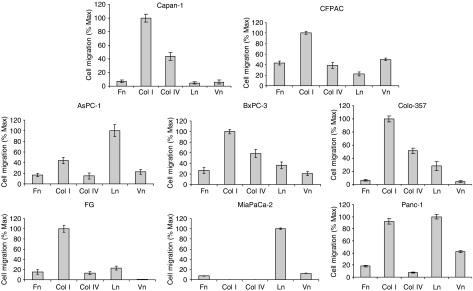
Type I collagen promotes maximal pancreatic cancer cell migration. Pore polycarbonate membrane filters (8 *μ*m) were coated with either type I collagen, type IV collagen, fibronectin, laminin, or vitronectin, at 5 *μ*g ml^−1^. Lower chambers of the modified Boyden chamber were filled with 30 *μ*l well^−1^ of serum-free DMEM supplemented with 1 mg ml^−1^ BSA, the coated filters were then placed on top of the lower chambers, and the upper chambers were secured in place. Upper chambers were filled with 5 × 10^4^ FG, AsPC-1, MiaPaCa-2, BxPC-3, or CFPAC cells that were serum-starved 24 h prior to assay, in 50 *μ*l of the same media described above. The entire apparatus was then incubated for 24 h at 37°C. After the incubation period, the filters were fixed in methanol and stained with 0.5% toluidine blue in 3.7% formaldehyde. Excess stain was washed away with water, the attached cells on the upper side of the filters were mechanically removed using wet, cotton-tipped applicators, and the migratory cells on the underside of the filters were quantitated by counting four high-powered fields (× 100 magnification) per well using an inverted light microscope (Olympus BH 2). Results presented represent the mean±s.e.m. from two independent experiments with at least three replicates per substrate for each cell line. 100%=maximal cell number for each cell line and each set of ECM proteins.

We next examined all of our cell lines for haptokinetic cell migration on the ECM. As opposed to chemotaxis, where cell migration is stimulated by a growth factor gradient, haptokinetic migration reflects the natural ability of the substrate to promote cell movement. Figure 3 demonstrates that type I collagen promotes maximal haptokinesis in all the SMAD4-mutated cell lines, including Capan-1, CFPAC, BxPC-3, Colo-357, and FG, cells. Interestingly, MiaPaCa-2, AsPC-1, and Panc-1 cells, which exhibit wild-type SMAD4, and exhibit only moderate cell adhesion on laminin (Figure 1), migrated maximally on that substrate. Panc-1 and AsPC-1 cells also showed significant migration on type I collagen, whereas MiaPaCa-2 cells exhibited no haptokinesis on the substrate. Vitronectin and fibronectin promoted relatively weak haptokinesis on all cell lines tested, although the two substrates promoted very strong cell adhesion in some cases (Figure 1). Relative to the other cell lines tested, BxPC-3 cells exhibited the most haptokinesis followed by Panc-1 and CFPAC, regardless of substrate (unpublished observations). Taken together, these results demonstrate that type I collagen promotes a phenotype that is consistent with malignancy in the majority of our pancreatic cancer cell lines, as defined by maximal cell adhesion, proliferation, and migration, relative to the other ECM proteins tested. These data also identify laminin as a strong migratory substrate in those cell lines lacking an SMAD4 mutation (AsPC-1, MiaPaCa-2, and Panc-1).

### The collagen-binding, *α*_2_*β*_1_ integrin mediates the malignant phenotype in pancreatic cancer cell lines

**Figure 4:**
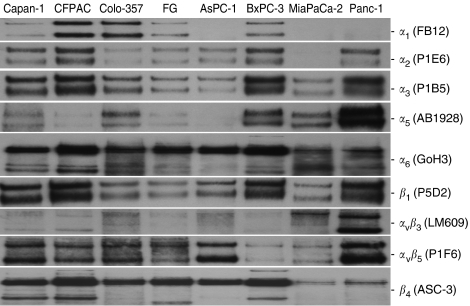
Integrin, E-cadherin, and *β*-catenin expression in pancreatic cancer cell lines. Immunoprecipitations were conducted using 4 *μ*g anti-integrin antibodies and 400 *μ*g cell-surface-biotinylated extracts from pancreatic cancer cell lines as described in Materials and Methods. Immunoprecipitates were separated on 12% Nu–PAGE gels under nonreducing conditions, and protein bands were visualised using streptavidin–HRP and chemiluminescence. Autoradiograms for each particular integrin subunit or integrin heterodimer for each cell line are indicated in the right-hand margin.

Integrins mediate the adhesion of cells to the ECM (Ruoslahti *et al*, 1994), and we have recently demonstrated in a limited study with FG cells that the *α*_2_*β*_1_ integrin mediates type I collagen adhesion and proliferation (Grzesiak *et al*, 2004, 2005a). Presently, we conducted IPs of all eight cell lines for relative expression of the *α*_1_, *α*_2_, *α*_3_, *α*_5_, *α*_6_, *β*_1_, and *β*_4_ integrin subunits, and the *α*_v_*β*_3_ and *α*_v_*β*_5_ integrins. Figure 4 demonstrates that the *α*_2_ integrin subunit is expressed by all of our pancreatic cancer cell lines, except for MiaPaCa-2 cells, which do not attach, proliferate, or migrate on types I or IV collagen under serum-free conditions (Figures 1, 2 and 3). Interestingly, Figure 4 also demonstrates that the collagen-binding *α*_1_ integrin subunit is expressed in all cell lines with TGF*β*_1_-activating SMAD4 mutations, including Capan-1, CFPAC, Colo-357, FG, and BxPC-3, but not in those cells expressing wild-type SMAD4, including AsPC-1, MiaPaCa-2, and Panc-1. All cell lines appear to be immunoreactive to varying degrees for the *α*_3_, *α*_5_, *α*_6_, *β*_1_, and *β*_4_ integrin subunits, as well as the *α*_v_*β*_3_ and *α*_v_*β*_5_ integrins. These integrin expression results are generally consistent with relative strength of adhesion and migration, BxPC-3, CFPAC, and Panc-1 cells demonstrate the strongest overall adhesion, migration, and integrin expression.

**Figure 5:**
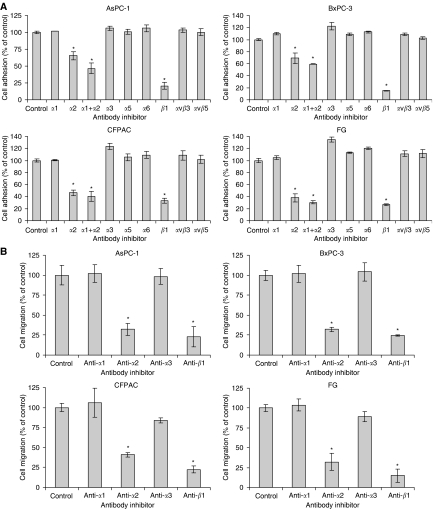
The *α*_2_*β*_1_ integrin mediates pancreatic cancer cell adhesion and haptokinetic migration on type I collagen. Inhibition of AsPC-1, BxPC-3, CFPAC, and FG cell adhesion and migration assays were conducted using 96-well plates or 8 *μ*M polycarbonate membranes coated with 5 *μ*g ml^−1^ type I collagen as described in Materials and Methods. Function-blocking monoclonal antibodies directed against the indicated integrins or integrin subunits were added at final concentrations ranging between 25 and 50 *μ*g ml^−1^ as described in Materials and Methods for each antibody and each cell line. (**A**) Cell adhesion and (**B**) haptokinetic cell migration.

As collagen-binding integrins have been shown to include *α*_1_*β*_1_, *α*_2_*β*_1_, and *α*_3_*β*_1_, depending on the cell type (Ruoslahti, 1991), and because these integrins are variously expressed by all of our cell lines, we conducted inhibition of adhesion and migration assays on type I collagen using function-blocking monoclonal antibodies directed against specific integrin subunits. Figure 5A and B demonstrate clearly that pancreatic cancer cell adhesion and migration on type I collagen is mediated exclusively by the *α*_2_*β*_1_ integrin, and that neither the *α*_1_, *α*_3_, *α*_5_, or *α*_6_ integrin subunits, nor the *α*_v_*β*_3_ or *α*_v_*β*_5_ integrins appear to be involved in the adhesive interactions between AsPC-1, BxPC-3, CFPAC, or FG pancreatic cancer cells and type I collagen. Immunoprecipitation studies shown in Figure 4 and previous studies by us and other laboratories (Cheresh and Spiro, 1987; Wayner and Carter, 1987; Sonnenberg *et al*, 1988; Wayner *et al*, 1988, 1993; Grzesiak *et al*, 1992, 2005a; Fabbri *et al*, 1996) demonstrate that the monoclonal antibodies used for the type I collagen inhibition studies are functional.

## DISCUSSION

In the present study, we characterised the adhesion, proliferation, and haptokinetic cell migration, as well as the relative integrin expression profiles of eight pancreatic cancer cells lines comprised of all three tumour grades, and derived from primary tumours, liver and lymph node metastases, and ascites. We tested physiologically relevant ECM components from the stroma, including type I collagen, fibronectin, and vitronectin, as well as the basement membrane proteins, type IV collagen and laminin, for their ability to promote a malignant phenotype in pancreatic cancer cell lines, as defined by increased adhesion, proliferation, and migration.

The clear indication that most of our pancreatic cancer cell lines attach, proliferate, and migrate maximally on type I collagen compared to the other ECM proteins examined is not surprising, in that the hallmark desmoplastic reaction associated with pancreatic adenocarcinoma, including upregulated type I collagen expression, has been appreciated for some time now (Mollenhauer *et al*, 1987; Lohr *et al*, 1994; Shimoyama *et al*, 1995; Tani *et al*, 1997; Kuehn *et al*, 1999; Linder *et al*, 2001; Tempia-Caliera *et al*, 2002). Recent analyses using microarray and serial analysis of gene expression technology confirm those studies and point out the strong expression of many other ECM genes in pancreatic cancer as well (Iacobuzio-Donahue *et al*, 2003a, 2003b; Binkley *et al*, 2004; Stein *et al*, 2004).

There is growing evidence that the desmoplastic response associated with pancreatic cancer is representative of dysregulated normal injury repair processes that include TGF*β*_1_-stimulated expression of type I collagen and other ECM proteins (Menke and Adler, 2002). In fact, it has been shown *in vitro* that TGF*β*_1_ upregulates type I collagen expression in pancreatic cancer (Lohr *et al*, 2001). Very recent studies from two independent laboratories also provide supporting data that type I collagen, in particular, plays an active role *in vitro* and *in vivo* in the pathophysiology of pancreatic cancer (Armstrong *et al*, 2004; Bachem *et al*, 2005). Specifically, these studies demonstrated *in vitro* that pancreatic cancer cell lines stimulated the production of type I collagen from adjacent stellate cells, which resulted in increased cancer cell proliferation and resistance to chemically induced apoptosis. *In vivo*, these studies also showed that type I collagen expression was closely associated with both the cancer and stellate cells, and that introduction of stellate cells along with tumour cells increased tumorigenesis in nude mice. Our present results are the first to indicate that even though there are several potential integrin receptors expressed by our pancreatic cancer cell lines, the *α*_2_*β*_1_ integrin exclusively mediates type I collagen adhesion, proliferation, and migration.

The previous demonstrations that type I collagen is clearly upregulated (Mollenhauer *et al*, 1987; Lohr *et al*, 1994; Shimoyama *et al*, 1995; Tani *et al*, 1997; Kuehn *et al*, 1999; Linder *et al*, 2001; Tempia-Caliera *et al*, 2002; Iacobuzio-Donahue *et al*, 2003a, 2003b; Binkley *et al*, 2004; Stein *et al*, 2004), and mediates a malignant phenotype in pancreatic cancer *in vivo* (Armstrong *et al*, 2004; Bachem *et al*, 2005), correlates directly with our present *in vitro* observations, and are further extended by our demonstration that the *α*_2_*β*_1_ integrin specifically regulates this type I collagen-mediated phenotype in multiple pancreatic cancer cell lines. These observations collectively suggest that targeting the *α*_2_*β*_1_ integrin may have therapeutic value in the treatment of pancreatic cancer *in vivo*. In support, inhibition of the *α*_2_*β*_1_ integrin with function-blocking monoclonal antibodies inhibited human osteosarcoma cell (HOS) tumour growth in SCID mice (Miura *et al*, 2005). In another study, OCUM-2MD3, a human scirrhous gastric carcinoma cell line overexpressing *α*_2_*β*_1_ and *α*_3_*β*_1_ integrins, produced peritoneal dissemination in nude mice compared to control cells, and its invasive ability was significantly decreased following the addition of *α*_2_*β*_1_ or *α*_3_*β*_1_ integrin antibodies (Nishimura *et al*, 1996).

Our present results also identify two distinct and previously unknown phenotypes that correlate directly with the presence or absence of TGF*β*_1_-activating SMAD4 mutations (see Table 1). In the five pancreatic cancer cell lines with SMAD4 mutations, including Capan-1, CFPAC, Colo-357, FG, and BxPC-3, type I collagen promotes maximal adhesion, migration, and proliferation. Regarding integrin expression, these same five cell lines express the collagen-binding *α*_1_*β*_1_ integrin. In the three cell lines expressing wild-type SMAD4, including AsPC-1, MiaPaCa-2, and Panc-1, maximal migration occurs on laminin and there is a distinct absence of the *α*_1_*β*_1_ integrin. These results are in agreement with previous results indicating that Panc-1 cells are negative and that BxPC-3 cells are positive for the *α*_1_ integrin subunit by FACS analyses (Lohr *et al*, 1996). It is intriguing that TGF*β*_1_ treatment of the NR4 hepatocyte cell line increased the expression of *α*_1_ integrin subunit mRNA (Serra *et al*, 1994), directly correlating with our findings that cell lines with TGF*β*_1_-activating SMAD4 mutations express the *α*_1_*β*_1_ integrin. Interestingly, while the *α*_1_*β*_1_ integrin is only expressed in cell lines containing SMAD4 mutations, the function-blocking monoclonal antibody against *α*_1_ had no effect on pancreatic cancer cell adhesion or migration on type I collagen either alone or in combination with the *α*_2_ function-blocking monoclonal antibody. Our data indicate that only the *α*_2_*β*_1_ integrin mediates type I collagen adhesion, migration, and proliferation in pancreatic cancer cell lines.

Immunoprecipitation results indicate that seven of the eight pancreatic cancer cell lines express the collagen-binding *α*_2_*β*_1_ integrin. While expression of the *α*_2_ integrin subunit has been demonstrated previously for most of these cell lines (Lohr *et al*, 1996; Sawai *et al*, 2001; Miyamoto *et al*, 2004), to our knowledge, this is the first demonstration that the CFPAC cell line is also *α*_2_ integrin subunit positive. These results are in agreement with reports using other pancreatic cancer cell lines, including PC-2, -3, and -44, PaTu 8988S, HPAF, Capan-2, PaTu 8902, and PaTu-2 cells, which also demonstrated type I collagen adhesion and *α*_2_ integrin subunit expression (Mollenhauer *et al*, 1987; Weinel *et al*, 1992). Our present results extend those previous studies by demonstrating, in multiple cell lines, the relative strength of type I collagen adhesion, proliferation, and migration compared to the other physiologically relevant ECM proteins tested in these studies. These results also rule out a contribution from the *α*_1_*β*_1_ integrin in mediating tumour cell adhesion to type I collagen in pancreatic cancer.

In agreement with previous studies, our results also indicate that MiaPaCa-2 cells do not attach to types I or IV collagen, and they do not express the *α*_2_ integrin subunit (Sawai *et al*, 2001; Miyamoto *et al*, 2004). In fact, it has been recently shown that attempts to grow MiaPaCa-2 cells on type I collagen substrates resulted in apoptosis (Vaquero *et al*, 2003). Our results extend these early findings, demonstrating for the first time that MiaPaCa-2 cells are negative for *α*_1_ integrin subunit expression as well. Additionally, we demonstrate that MiaPaCa-2 cells attach and proliferate strongly on vitronectin and fibronectin, and are very migratory on laminin.

Adhesion of pancreatic cancer cell lines to vitronectin yielded the most wide-ranging results, with MiaPaCa-2, Capan-1, AsPC-1, and Panc-1 exhibiting strong adhesion and proliferation (MiaPaCa-2 and AsPC-1), and CFPAC, FG, Colo-357, and BxPC-3 exhibiting weak adhesion and proliferation (CFPAC, FG, and BxPC-3). It is also interesting that three of the four cell lines demonstrating strong vitronectin adhesion, including Panc-1, MiaPaCa-2, and AsPC-1, also express wild-type SMAD4.

In summary, these studies using a broad panel of pancreatic cancer cell lines strongly implicate the *α*_2_*β*_1_ integrin in the promotion of the malignant phenotype on type I collagen in pancreatic cancer, as defined by increased adhesion, proliferation, and migration. These results further identify the *α*_2_*β*_1_ integrin as a potential therapeutic target in the treatment of this devastating disease. These studies also identify dramatic differences in integrin expression and ECM substrate preferences in pancreatic cancer cell lines expressing wild-type *vs* mutated SMAD4. Future studies will aim at the inhibition of pancreatic cancer growth, progression, and metastasis in our orthotopic mouse models using function-blocking monoclonals against *α*_2_*β*_1_ integrin function.

## Figures and Tables

**Table 1:** Origin, grade, and genetic mutations of pancreatic cancer cell lines (Peng *et al*, 2002; Sipos *et al*, 2003; Monti *et al*, 2004).

**Cell line**	**Origin**	**Grade**	**K-ras**	**p53**	**p16^ink4A^**	**DPC4/Smad4**
Capan-1	Liver metastasis	1	mut	mut	mut	mut
CFPAC	Liver metastasis	1	mut	mut	mut	mut
Colo-357	Lymph node metastasis	2	mut	w.t.	w.t.	mut
FG	Lymph node metastasis	2	mut	w.t.	w.t.	mut
AsPC-1	Ascites	2	mut	mut	Mut	w.t.
BxPC-3	Primary	2	w.t.	mut	mut	mut
MiaPaCa-2	Primary	3	mut	mut	mut	w.t.
Panc-1	Primary	3	mut	mut	mut	w.t.

mut=mutated; w.t.=wild type.
